# Efficacy and Safety of Allisartan Isoproxil/Amlodipine in Patients with Essential Hypertension: A Phase III, Multicenter, Double-Blind, Parallel-Group, Randomised study

**DOI:** 10.1038/s41371-025-01035-3

**Published:** 2025-06-17

**Authors:** Hongjie Chi, Xin Zhang, Shumei Ma, Gang Pan, Xiaojuan Lian, Yuanyuan Chen, Yan Chen, Hao Tang, Zichen Liu, Peng Mi, Xiangmin Lin

**Affiliations:** 1 Department of Cardiology, Beijing Chaoyang Hospital, Capital Medical University, Beijing, China; Heart Center and Beijing Key Laboratory of Hypertension, Beijing Chaoyang Hospital, Capital Medical University, 8 Gongren Tiyuchang Nanlu, Chaoyang District, Beijing, China; 2 The First Affiliated Hospital of Baotou Medical College, No.41 Linyin Road, Kundulun District, Baotou City, Inner Mongolia, China; 3 Shengjing Hospital of China Medical University, No.36 Sanhao Street, Heping District, Shenyang, Liaoning Province China; 4 Yueyang Central Hospital, 39 Dongmaoling Road, Yueyang City, Hunan Province China; 5 Shenzhen Salubris Pharmaceuticals Co., LTD, Beijing, China

**Keywords:** Stroke, Clinical trials

## Abstract

This multicenter, double-blind, parallel-group, randomised controlled phase III study evaluated the efficacy and safety of the Allisartan Isoproxil 240 mg /Amlodipine 5 mg (ALI/AML) combination compared with ALI 240 mg monotherapy in patients with mild-to-moderate essential hypertension. Patients aged 18 to 70 years with mean sitting systolic blood pressure (msSBP) between 140 and <180 mmHg and mean sitting diastolic blood pressure (msDBP) between 90 and <110 mmHg were randomised 1:1 to receive ALI/AML or ALI once-daily for 12 weeks after a 4-week treatment with ALI, followed by an open-label extension period with ALI/AML up to week 52. A total of 199 patients were randomised (ALI/AML: n = 99, ALI: n = 100) with 169 completing the study. Baseline characteristics were comparable between groups. After 12 weeks of randomisation, the reduction in msSBP (primary endpoint) was significantly greater in the ALI/AML group vs the ALI group (−18.3 vs. −9.3 mmHg, *p* < 0.001). Reductions in msDBP (−6.0 vs. −1.9 mmHg, *p* < 0.001) and 24-hour mean ambulatory systolic/diastolic blood pressure (−19.9/−10.1 vs. −6.9/−4.2 mmHg) were more pronounced in the ALI/AML group. Additionally, a greater proportion of patients achieved the BP response and target office BP in the ALI/AML group compared to the ALI group (53.6% vs. 25.5%, *p* < 0.001; 40.2% vs. 20.4%, *p*=0.0026). AML/ALI combination was generally safe and well tolerated, with sustained efficacy up to 52 weeks. The study concluded that ALI/AML offers a convenient, single-pill option for effective BP reduction in hypertensive patients.

## Introduction

Hypertension, defined as chronic high blood pressure, is a critical risk factor strongly associated with the incidence and mortality of cardiovascular and cerebrovascular diseases [1, 2]. Elevated systolic and diastolic blood pressure levels are well-established predictors of increased risks for cardiovascular disease, angina, myocardial infarction, heart failure, stroke, peripheral artery disease, and abdominal aortic aneurysm, each of which is independently significant. Globally, over 1 billion individuals currently live with uncontrolled hypertension, a number projected to rise to 1.5 billion by 2030 [3]. This alarming trend is driven by an aging population and escalating exposure to lifestyle risk factors, including poor dietary habits—marked by high sodium and low potassium intake—and sedentary behavior, leading to suboptimal blood pressure control among many hypertensive patients. In China, awareness, treatment, and control rates remain disappointingly low [4–6], with an estimated 245 million hypertensive patients in 2022 [7]. The escalating burden of hypertension and its associated cardiovascular and cerebrovascular complications poses a significant and growing public health concern.

Hypertension management strategies encompass lifestyle modifications, pharmacological interventions, and device-based therapies [8, 9]. However, pharmacological therapy remains the cornerstone of effective blood pressure control [8, 9]. Commonly used antihypertensive medications comprise calcium channel blockers (CCBs), angiotensin-converting enzyme inhibitors (ACEIs), angiotensin II receptor blockers (ARBs), diuretics, and β-blockers, alongside fixed-dose combinations of these agents [8, 10]. The Single-Pill Combination (SPC) approach represents an innovative strategy in combination therapy. System reviews have demonstrated that SPCs, which combine multiple components, improve treatment adherence and drug persistence compared to non-fixed combinations [11, 12]. Current European [13, 14], Chinese [8], and American [15] guidelines recommend the combination of ACEI/ARB with CCB as a preferred treatment strategy.

ALI, an ARB-class medication, has been positively evaluated for its antihypertensive efficacy in patients with mild-to-moderate hypertension [16]. It has been shown to mitigate renal impairment associated with hypertension and exhibits exceptional tolerability among essential hypertensive populations [17, 18]. AML, a CCB, exerts its effectiveness by inhibiting voltage-dependent L-type calcium channels [19]. It manifests protective effects on endothelial cell function and anti-atherosclerotic properties [20]. Its efficacy in blood pressure management is well-established in clinical practice. AML is often used in conjunction with other antihypertensive medications, such as ACEIs, ARBs, and diuretics [8, 19]. Systematic reviews have shown that ACEI/ARB combinations significantly enhance metabolic and renal functions in hypertensive patients, surpassing the benefits of other dual or triple drug regimens [21].

The objective of this study was to assess the efficacy and safety of ALI/AML Tablets in patients with essential hypertension uncontrolled after 4 weeks of monotherapy with ALI.

## Materials and Methods

### Study Design

This Phase III, 52-week, multicenter, randomised, double-blind, parallel-controlled trial was conducted across 42 sites in China from January 2021 to February 2023. Patients with mild-to-moderate essential hypertension were enrolled if they were either treatment-naïve, had previously received other antihypertensive agents for ≥ 2 weeks, or had irregular treatment with ALI, and they were initially treated with ALI (240 mg/day) for a 4-week run-in period. Those with uncontrolled blood pressure (msSBP/msDBP≥140 mmHg/90 mmHg) subsequently entered a 12-week double-blind treatment period. Participants on a stable ALI regimen (240 mg/day) for ≥4 weeks with uncontrolled BP were directly randomised into the 12-week double-blind period. Following the double-blind treatment period, all participants entered the open-label treatment period, receiving the ALI/AML combination treatment for a 52-week assessment of long-term safety and tolerability.

Randomisation was performed using a stratified block design (block size = 4) with the randomisation list generated using SAS (version 9.4). Eligible patients were randomised in a 1:1 ratio, stratified by baseline office SBP (<160 or ≥160 mmHg) to receive either ALI 240 mg plus placebo matching the ALI/AML combination, or ALI/AML(240 mg/5 mg) along with a placebo matching ALI.

Blinding was maintained through standardized packaging. Each participant was provided with a large box containing a 5-week supply of medication, subdivided into five smaller boxes (each for 1 week). Each smaller box contained seven daily dose sachets. For ALI/AML group, each sachet contained one ALI/AML tablet (240 mg/5 mg) and one ALI-matching placebo tablet. For the ALI monotherapy group, each sachet contained one ALI tablet (240 mg) and one ALI/AML combination–matching placebo tablet. Identical packaging, color, appearance, smell, and labeling were employed across groups to ensure proper blinding.

For the ABPM sub-study, 40 participants were planned to be recruited. The eligibility criteria required participants to have uncontrolled blood pressure after 4 weeks of AML monotherapy, with a 24-hour mean ambulatory BP of ≥130/80 mmHg.

The study was approved by ethics committees of all participating centers and was conducted in accordance with the Declaration of Helsinki and the Good Clinical Practice guidelines. The trial was registered with ClinicalTrials.gov (NCT06465264).

### Study Population

Patients were eligible for the study in the following conditions:

Primary inclusion criteria:

1. Patients aged 18 to 70 with mild-to-moderate essential hypertension;
2. Untreated patients (either newly diagnosed or with a history of hypertension who have not taken any antihypertensive medications for ≥ 2 weeks before screening) with msSBP 150 to < 180 mmHg and msDBP < 110 mmHg; patients with a history of irregular ALI treatment (240 mg once daily for < 4 weeks) or those who had missed doses for ≥ 5 days within the last 4 weeks, with msSBP 140 to <180 mmHg and msDBP < 110 mmHg; patients who had been on other antihypertensive therapies for ≥ 2 weeks with msSBP 140 to <180 mmHg and msDBP < 110 mmHg, deemed suitable for a switch to ALI 240 mg /day by the treating clinician; and patients who had been on ALI 240 mg/day for ≥ 4 weeks with msSBP 140 to <180 mmHg and msDBP < 110 mmHg;
3. Patients with msSBP 140 to < 180 mmHg and msDBP < 110 mmHg before randomisation;
4. Participants in the ABPM substudy with a 24-hour mean ambulatory blood pressure of ≥130/80 mmHg after 4 weeks of monotherapy.

Key exclusion criteria

1. Secondary hypertension;
2. MsSBP≥ 180 mmHg and/or msDBP≥ 110 mmHg, or a history of hypertensive emergencies or urgencies;
3. History of New York Heart Association (NYHA) Class III or IV heart failure, acute coronary syndrome, percutaneous coronary intervention, or other severe cardiac conditions such as cardiogenic shock, moderate-to-severe valvular heart disease, second-or third-degree atrioventricular block, bradycardia with a heart rate below 50 beats per minute, or severe arrhythmias within 6 months;
4. History of severe cerebrovascular events including hypertensive encephalopathy, cerebrovascular injury, stroke, or transient ischemic attack within the past 6 months;
5. Large aneurysm, aortic dissection, or dissecting aneurysm;
6. Renal artery stenosis or severe renal insufficiency;
7. Known allergies or intolerance to study drugs (such as angioedema);
8. Active viral hepatitis (including hepatitis B and hepatitis C), severe liver disease, or liver dysfunction;
9. Abnormal laboratory findings, including fasting blood glucose ≥ 11 mmol/L, creatinine > 1.5 times the upper limit of normal (ULN), potassium > 5.5 mmol/L, alanine aminotransferase (ALT) or aspartate aminotransferase (AST) > 2.5 times ULN, or total bilirubin (TBIL) > 2 times ULN. Additional details regarding eligibility criteria are provided in the Supplementary Material. All participants provided written informed consent before being included in the study.

### Study Endpoint

#### Efficacy

##### Endpoints

Primary Endpoint

Change in msSBP at Week 12 post-randomisation.

Secondary Endpoints

1. Change in msDBP at Week12 post-randomisation;
2. Changes in msSBP and msDBP at Weeks 4 and 8 after randomisation;
3. Proportion in responders (msSBP/msDBP<140/90 mmHg, or a reduction in msSBP >20 mmHg and/or mean msDBP >10 mmHg) at Week12 after randomisation;
4. Target BP rates (msSBP/msDBP<140/90 mmHg) at Week 4, Week 8, Week 12, and Week 32 after randomisation as well as at the end of treatment.

Exploratory endpoints

Changes in 24-hour, morning, daytime, and nighttime mean ambulatory SBP/DBP at Week 12 after randomisation.

##### BP Measurements

Office BP measurements were conducted during the screening period, run-in period, and at Weeks 0, 4, 8, 12, 22, 32, 42, and 52 post- randomisation, following the hypertension management guidelines (*Verification Regulation of Non-invasive Automatic Sphygmomanometer JJG 692-2010*). The validated and calibrated upper-arm electronic blood pressure monitor (UA-651BLE-W, A&D Electronics Co., Ltd. Shenzhen, China) was utilized. In the morning, Office BP was measured twice at 1-2 minute intervals, with the mean value recorded; an additional measurement was performed if the difference between the two measurements of msSBP and/ or msDBP was ≥ 5 mmHg. Orthostatic BP should be measured after the last SBP measurement, with a one-minute delay after transitioning to an orthostatic position. Orthostatic hypotension was defined as a SBP reduction of ≥20 mmHg and/or a DBP reduction of ≥10 mmHg, irrespective of hypotension symptoms.

ABPM was also conducted during the screening, run-in periods, and at Week 12 post-randomisation using the AND TM-2430 device, following hypertension management protocols (*Verification Regulation of Non-invasive Automated Sphygmomanometers JJG 692-2010*). Before ABPM, clinic BP was measured in both arms; the higher BP arm was used, if the difference was ≥10 mmHg, otherwise, the non-dominant arm was selected. ABPM readings were taken every 20 minutes during the daytime awakening hours and every 30 minutes during nighttime sleeping hours. For ABPM validity, a minimum of 20 daytime readings, 7 nighttime readings, and 70% of the anticipated 24-hour readings (with at least one reading per hour) were required. The early morning period was defined as the initial 2 hours post-awakening, based on the participant’s schedule. The “awakening” and “sleeping” times were determined through participant self-reports. Changes from baseline in the morning, daytime, and nighttime SBP/DBP were analyzed. 40 participants were planned for the ABPM analysis.

The Shuoyun Integrated Information Management System (1.6.2.210422, Shanghai Shuo Yun Information Technology Co., Ltd. Shanghai, China) was used for data collection and reporting of both ABPM and office BP measurements.

#### Safety

Safety assessments included adverse events (AEs) and serious adverse events (SAEs), clinical laboratory tests (hematology, serum biochemistry, urinalysis), vital signs (excluding BP), physical examinations, 12-lead electrocardiograms(ECGs), and other relevant parameters. AEs and SAEs were documented from the time of consent. The laboratory tests were conducted during the screening period, the run-in period, as well as at Weeks 12, 32, and 52. Vital signs were measured during the screening period, run-in period, as well as at Weeks 0, 4, 8, 12, 22, 32, 42, and 52. Physical examinations were conducted during the screening period, run-in period, as well as at Weeks 12 and 52. 12-lead ECGs were performed during the screening period, run-in period, as well as at Weeks 4, 8, 12, 22, 32, 42, and 52.

### Statistical Analysis

The sample size was calculated to detect a 5 mmHg difference in mean systolic blood pressure (msSBP) between ALI/AML and ALI, with 80% power, a standard deviation of 11 mmHg, and a one-sided significance level of 0.025 [22]. Accounting for a 20% dropout rate, 194 patients (97 per treatment arm) were required. Calculations were performed using PASS 16 software.

In this study, the intention-to-treat (ITT) analysis set includes all randomised subjects, regardless of whether they received the study medication. The full analysis set (FAS), based on the ITT principle, includes all subjects who were randomised, received at least one dose of the study drug during the double-blind period, and had at least one post-dose efficacy assessment. The PPS consists of subjects in the FAS who did not have any major protocol deviations and who provided data for the primary efficacy endpoint.

The primary efficacy endpoint analysis was analyzed using a mixed model for repeated measures (MMRM) on the FAS with supplementary analyses on the PPS. The model incorporated changes in msSBP at each visit during the double-blind treatment period as the dependent variable. The model included treatment group, time, and group-time interactions as fixed effects, and baseline msSBP as a covariate. The difference in least squares mean (LSM), 95% confidence intervals (CIs), and *P* values were calculated. Additionally, subgroup analyses based on randomisation stratification and other baseline characteristics were conducted.

The secondary endpoints of changes in msSBP and msDBP were evaluated with MMRM on FAS and PPS, and target BP rates or response rates at Week 12 post-randomisation were analysed using a logistic regression model. The target BP rates at Week 32 and at the end of treatment were analysed using descriptive statistics.

The baseline characteristics were summarized using descriptive statistics on the FAS, and the safety assessments were conducted on the safety set (SS). All statistical analyses were conducted using SAS version 9.4 software (SAS Institute, Inc.).

## Results

### Study Participants

**Fig. 1:**
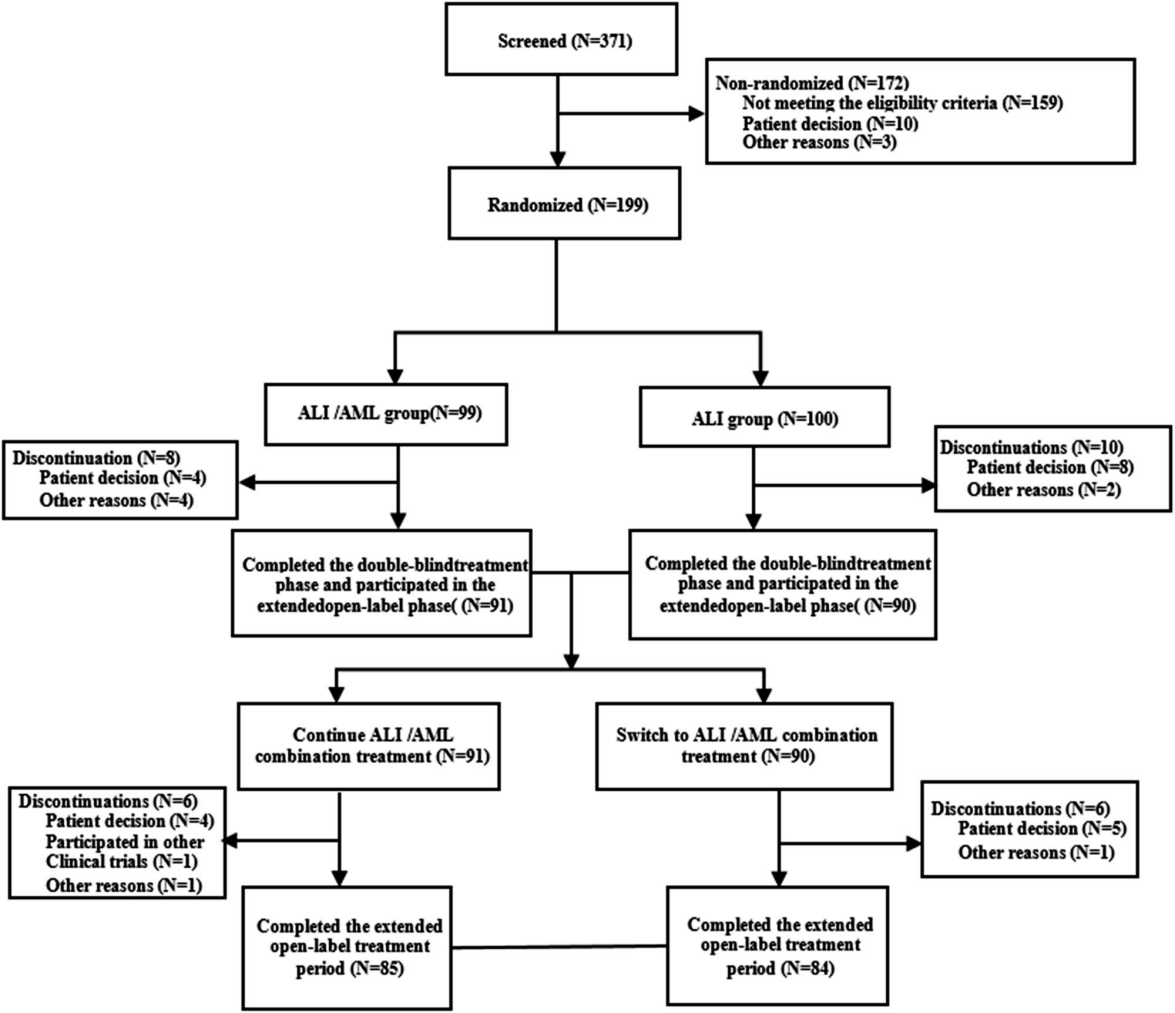
Patient disposition.

A total of 371 patients were screened, out of which 199 patients were randomised, while 172 patients were not. The reasons for non-randomisation included 159 patients who did not meet the eligibility criteria requiring an inadequate response to ALI 240 mg/day treatment, 10 patients discontinued before randomisation, and 3 patients for other reasons (1 for protocol violation, 2 for loss to follow-up). A total of 169 patients completed the study (Fig. 1). The FAS included 195 patients (97 in the ALI/AML group and 98 in the ALI group). The SS included 198 patients (98 in the ALI/AML group and 100 in the ALI group). Compliance was similar in both groups (98.0% vs. 97.9%) throughout the study.

The patients had a mean age of 57.2 years, with 58.5% males and 14.4% diabetic patients. Baseline msSBP/msDBP showed comparable values between groups (154.5/91.0 mmHg in ALI/AML vs. 154.8/88.9 mmHg in ALI), The baseline characteristics of the two groups were basically consistent and generally comparable (Table 1).

**Table 1:** Summary of demographic characteristics (FAS).

Index	Statistics	ALI/AML group (N = 97)	ALI group (N = 98)	Total (N = 195)
Gender, n (%)	Female	43 (44.3)	38 (38.8)	81 (41.5)
Race, n (%)	Han ethnic	96 (99.0)	98 (100)	194 (99.5)
	Other	1 (1.0)	0	1 (0.5)
Age (years)	Mean (SD)	56.3 (9.00)	58.0 (7.83)	57.2 (8.45)
Age categorization, n (%)	<65	74 (76.3)	74 (75.5)	148 (75.9)
	≥65	23 (23.7)	24 (24.5)	47 (24.1)
BMI (kg/m^2^)^a^	Mean (SD)	25.98 (2.51)	26.16 (2.48)	26.07 (2.49)
Baseline msSBP	Mean (SD)	154.5 (10.20)	154.8 (8.64)	-
Baseline msDBP	Mean (SD)	91.0 (9.93)	88.9 (9.93)	-
Classification of baseline hypertension levels, n (%)^b^	Grade 1	55 (56.7)	56 (57.1)	111 (56.9)
	Grade2	42 (43.3)	42 (42.9)	84 (43.1)
Duration of hypertension (years), n (%)^c^	<1	23 (23.7)	19 (19.4)	42 (21.5)
	≥1 and <5	26 (26.8)	30 (30.6)	56 (28.7)
	≥5 and <10	27 (27.8)	21 (21.4)	48 (24.6)
	≥10	21 (21.6)	28 (28.6)	49 (25.1)
Uric Acid (μmol/L)	Mean (SD)	339.5 (103.0)	349.4 (100.2)	-
History of hyperuricemia, n (%)	YES	16 (16.5)	14 (14.3)	30 (15.4)
	NO	81 (83.5)	84 (85.7)	165 (84.6)
History of diabetes mellitus, n (%)	YES	11 (11.3)	17 (17.3)	28 (14.4)
	NO	86 (88.7)	81 (82.7)	167 (85.6)

^a^BMI (kg/m^2^) = body weight (kg)/ height^2^ (m^2^).

^b^Grade 1 hypertension is defined as msSBP of 140-159 mmHg or msDBP of 90-99 mmHg, and does not meet the grade 2 criteria; Grade 2 hypertension: msSBP is 160-179 mmHg or msDBP is 100-109 mmHg.

^c^Duration of hypertension (years) = (date of informed consent - time of first diagnosis of hypertension + 1)/365.25.

### Office BP Measurements

**Fig. 2:**
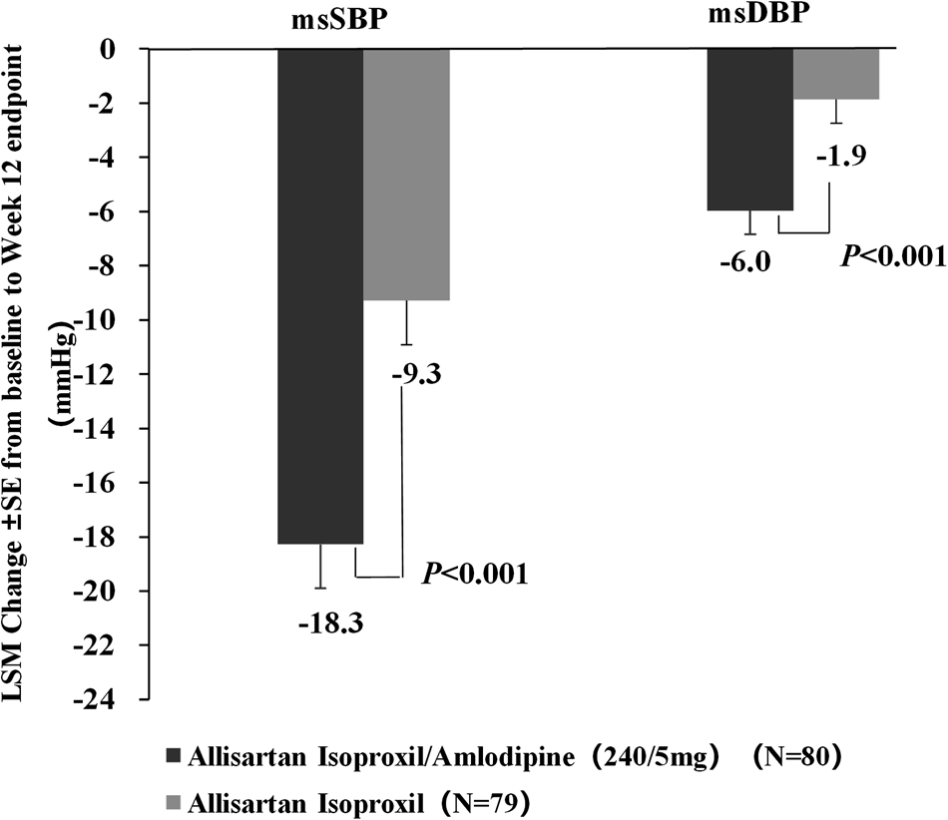
Comparison of change from baseline to Week 12 in msSBP and msDBP between ALI/AML (240/5 mg) and ALI (240 mg) groups(MMRM). LSM indicates least square mean.

**Fig. 3:**
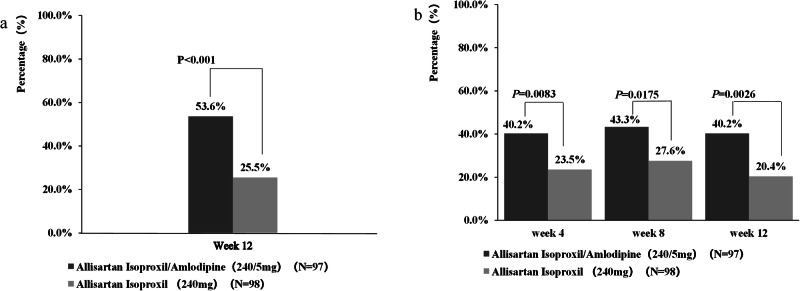
Proportion of responders and target BP rates in the double-blind period (Logistic Regression). **a** Proportion of responders (msSBP/msDBP<140/90 mmHg, or a reduction in msSBP >20 mmHg and/or mean msDBP >10 mmHg). **b** Target BP rates (msSBP/msDBP<140/90 mmHg).

At Week 12, the ALI/AML group demonstrated superior efficacy compared to the ALI group, with significantly greater reductions in msSBP (−18.3 vs. −9.3 mmHg; between-group difference: −9.0 mmHg, 95% CI: −13.53 to −4.45, *p* < 0.001) and msDBP (−6.0 vs.−1.9 mmHg; between-group difference: −4.1 mmHg, 95% CI: −6.5 to −1.7, *p* < 0.001) (Fig. 2). Additionally, the ALI/AML group exhibited significantly greater proportions of responders and target BP rates compared to the ALI group, with consistent results across FAS and PPS analyses (Fig. 3).

During open-label treatment, both groups received ALI/AML. By the end of treatment, msSBP/msDBP reductions reached −21.7/-4.8 mmHg (ALI/AML) versus −21.5/−5.2 mmHg (ALI-switched). The target BP rates in the ALI/AML group and the ALI -switched group were 67.4% vs. 65.1% at Week 32 and 53.5% vs. 54.8% at the end of treatment, respectively.

### Subgroup Analysis

**Fig. 4:**
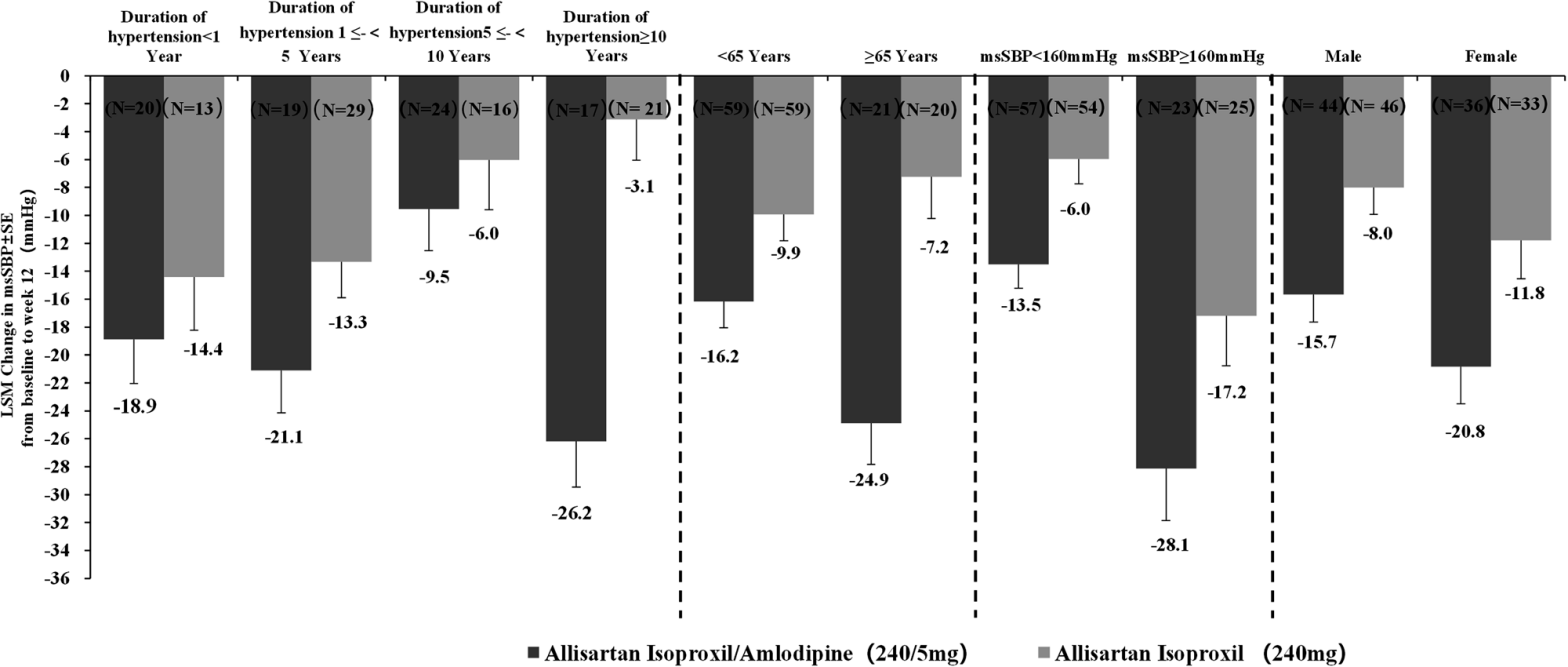
Changes in msSBP from baseline to Week 12 in different subgroups between ALI/AML (240/5 mg) and ALI (240 mg) groups. LSM indicates least square mean(MMRM).

Subgroup analyses were performed to evaluate the primary efficacy endpoint at Week 12 based on the following baseline factors: gender (male vs. female), age (≥65 years vs. <65 years), duration of hypertension (<1 year, ≥1 year and <5 years, ≥5 years and <10 years, ≥10 years), and baseline hypertension levels (msSBP≥160 mmHg vs. <160 mmHg). These analyses demonstrated that the ALI/AML group consistently achieved greater msSBP reductions compared to the ALI group across all baseline factors, as shown in Fig. 4.

### Ambulatory BP Measurements

**Fig. 5:**
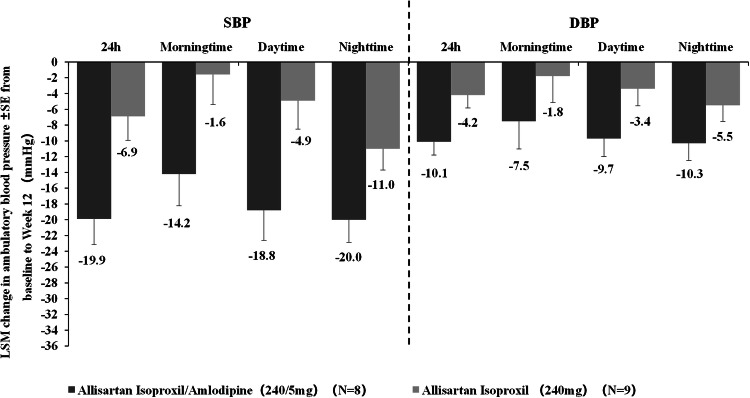
LSM change from baseline to Week 12 in ambulatory blood pressure between ALI/AML (240/5 mg) and ALI (240 mg) groups (ANCOVA). LSM indicates least square mean(MMRM).

**Fig. 6:**
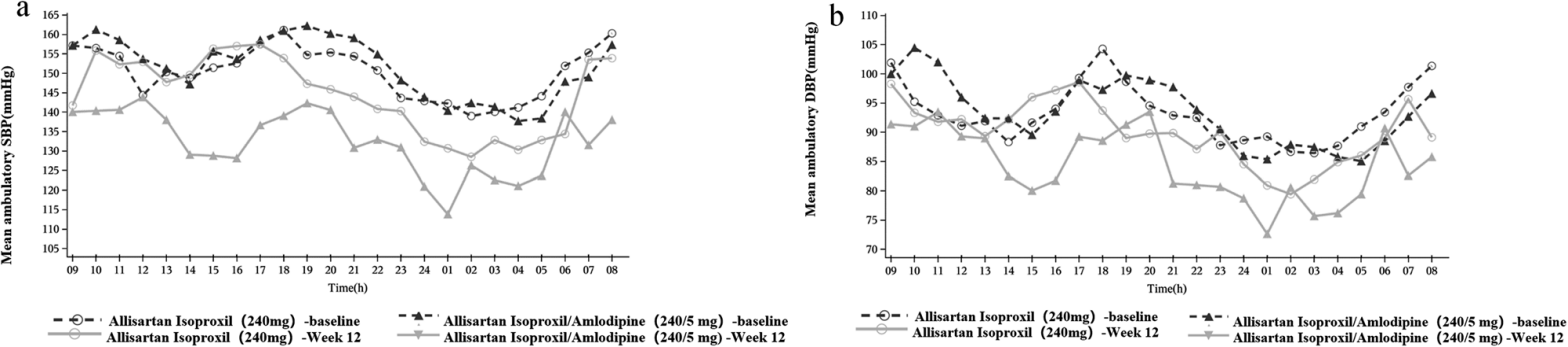
Postdosing hourly mean ambulatory SBP and DBP at Week 12 with Allisartan Isoproxil/Amlodipine vs. Allisartan Isoproxil groups. **a** Mean ambulatory SBP (n = 8). **b** Mean ambulatory DBP (n = 9).

In the ABPM analysis, 37 subjects were included (19 in the ALI/AML group and 18 in the ALI group). At Week 12, the ALI/AML group had significantly greater reductions in 24-hour mean systolic and diastolic BP compared to the ALI group (systolic: −19.9 vs. −6.9 mmHg; diastolic: −10.1 vs. −4.2 mmHg; both *p* < 0.05). The LSM reductions from baseline in daytime ambulatory BP were also greater in the ALI/AML group (systolic: −18.8 vs. −4.9 mmHg, *P* = 0.0193; diastolic: −9.7 vs. −3.4 mmHg, *P* = 0.0681). Similarly, nighttime ambulatory BP were reduced more in the ALI/AML group (systolic: −20.0 vs. −11.0 mmHg, *P* = 0.0396; diastolic: −10.3 vs. −5.5 mmHg, *P* = 0.1392). The ALI/AML group also showed more pronounced reductions in morning ambulatory BP compared to the ALI group (see Figs. 5 and 6).

### Safety

#### Summary of Adverse Events

In the double-blind period, adverse events (AEs) occurred in 19 (19.4%) patients in the ALI/AML group and 22 (22.0%) patients in the ALI group, with most AEs being mild. The most frequently reported AEs in the ALI/AML group were dizziness 2(2.0%) and arrhythmias 2(2.0%), while in the ALI group, dizziness 6(6.0%) and upper respiratory tract infection 4(4.0%) were more frequent (Table 2). Drug-related AEs occurred in 1.0% (1/100) of ALI/AML patients versus 6.0% (6/100) in the ALI group. In the ALI/AML group, the most frequent drug-related AEs were hyperlipidemia 1(1.0%), while in the ALI group, dizziness 3(3.0%), headache 2(2.0%) and electrocardiogram T-wave abnormalities 2(2.0%) were the most common.

**Table 2:** Incidence of adverse events in double-blind period(SS).

PT	ALI/AML group (N = 98)	ALI group (N = 100)
	n(%)	n(%)
Any AE^a^	19 (19.4)	22 (22.0)
Upper respiratory tract infection	1 (1.0)	4 (4.0)
Nasopharyngitis	1 (1.0)	2 (2.0)
Dizziness	2 (2.0)	6 (6.0)
Headache	0	3 (3.0)
Arrhythmia	2 (2.0)	0
Hyperglycemia	0	2 (2.0)
Abnormal T wave of ECG	0	2 (2.0)

^a^Only AEs that occurred in ≥2 subjects were listed.

In the open-label period, The overall incidence of AEs increased to 126(69.6%), with most events being mild or moderate in severity. The most common AEs were hyperuricemia 17(9.4%), COVID-19 15(8.3%;), hyperlipidemia 14(7.7%), and hypertriglyceridemia 13(7.2%). The incidence of drug-related AEs increased to 38(21.0%), with hypercholesterolemia 7(3.9%), blood glucose increased 6(3.3%) and hypertriglyceridemia 5(2.8%) being the most frequent.

#### Withdrawals Due to AEs

During the double-blind period, AEs leading to study withdrawal occurred in 2(2.0%) in the ALI/AML group(head discomfort and sudden death, each affecting 1 patient) and 1(1.0%) in the ALI group (dizziness). In the open-label period, 1 (0.6%) patient withdrew due to hypoesthesia and palpitations. None of the AEs leading to withdrawal were related to the study drugs .

#### SAEs

During the double-blind period, SAEs were reported in 2 (2.0%) patients in the ALI/AML group (herpes zoster in 1 patient and sudden death in 1 patient) and in 1 (1.0%) patient in the ALI group (atrial fibrillation). None of the SAEs during this period were considered related to the study drugs. During the open-label period, SAEs occurred in 9 (5.0%) patients, including 1 (0.6%) case of angina unstable related to the study drugs. No SAEs led to withdrawal.

#### Laboratory Findings

During the double-blind period, hematological and clinical chemistry parameters showed no obvious differences between treatment groups (Supplementary Table 1). Most patients maintained normal blood potassium levels (3.5-5.5 mmol/L), and no patients had sodium levels < 130 mmol/L. Baseline uric acid levels were 339.5 μmol/L in the ALI/AML group and 349.4 μmol/L in the ALI group (both below the hyperuricemia threshold of 420 μmol/L). At Week 12, the reductions in uric acid levels were 12.4 μmol/L in the ALI/AML and 8.6 μmol/L in the ALI groups, with the ALI/AML group showing a greater reduction. During the open-label period, most patients maintained normal blood potassium levels, and no patient had sodium levels <130 mmol/L.

## Discussion

This phase III, multicenter, double-blind, parallel-group, randomised clinical study demonstrated the efficacy and safety of SPC of ALI/AML tablets in patients with essential hypertension uncontrolled after 4 weeks of ALI monotherapy.

All baseline characteristics were generally comparable between the ALI/AML and ALI treatment groups. The ALI/AML combination therapy demonstrated superiority in reducing msSBP from baseline to Week 12 compared with ALI monotherapy. In addition, subgroup analyses, stratified by age, gender, duration of hypertension, and baseline blood pressure levels, consistently showed greater msSBP reductions in the ALI/AML group, aligning with the overall trends for the primary efficacy endpoint. Treatment adherence was high in both groups (98.0% vs 97.9%). The antihypertensive effect of ALI/AML was sustained for up to 52 weeks. By the study’s end, over 50% of patients in both groups achieved target office blood pressure, compared with a 40.2% rate in the ALI/AML group at Week 12. This improvement indicates sustained efficacy with continued treatment.

Hypertension is a major modifiable risk factor for cardiovascular disease [1, 2]. The results of the Blood Pressure Lowering Treatment Trialists’ Collaboration (BPLTTC) study indicate that a 5 mmHg reduction in SBP could reduce the risk of major adverse cardiovascular events (MACE) by 10%. Similarly, a 3 mmHg reduction in DBP lowers the risk of MACE in hypertensive patients of all ages [23]. In our study, at Week 4, the LSM changes in msSBP/msDBP were −16.1/-5.2 mmHg in the ALI/AML group and −6.9/-1.5 mmHg in the ALI group, with between-group differences of −9.3/−3.7 mmHg. By Week 12, the LSM changes were −18.3/-6.0 mmHg in the ALI/AML group, compared to −9.3/−1.9 mmHg in the ALI group, yielding difference of −9.0 /−4.1 mmHg.

ABPM is regarded as the optimal method for reliably assessing actual blood pressure. The average values over a 24-hour period, including daytime and nighttime periods, correlate more strongly with mortality and cardiovascular disease risk [24]. At Week 12, the ALI/AML combination group also demonstrated potent and sustained antihypertensive effects over 24 hours, with greater reductions in 24-hour, morning time, daytime, and nighttime mean ambulatory SBP/DBP compared to the monotherapy group, emphasizing the clinical efficacy of ALI/AML. Compared with daytime blood pressure, nighttime blood pressure is more closely associated with the risk of all-cause mortality and cardiovascular and cerebrovascular disease mortality [25]. Previous studies have shown that ALI has more advantages in nocturnal BP reduction among the ARBs, making it a preferred treatment for nocturnal hypertension [26, 27]. This study revalidated ALI’s advantage, with the ALI/AML combination demonstrating the property of greater nighttime blood pressure reduction compared to daytime, offering a novel treatment option for nocturnal hypertension.

ALI/AML demonstrated favorable safety and tolerability in hypertensive patients. During the double-blind period, the overall incidence of AEs was comparable between the treatment groups, with the majority of AEs being mild. The dizziness incidence in the ALI/AML group (2.0%) did not exceed that in the ALI monotherapy group (6.0%) and remained consistent with prior ALI studies (2.2%) [18].

Notably, no cases of hypotension or orthostatic hypotension were observed throughout the study. Additionally, peripheral edema, which is typically induced by CCBs through venous dilation, was not reported. This finding may be attributed to the complementary action of ARBs, like ALI, which counteract the edema-inducing effects of CCBs [28]. Hyperkalemia, a potential effect of Renin-Angiotensin-Aldosterone System inhibitors such as ALI [29], was not reported during the double-blind period, and its incidence was less than 1% (0.6%) during the open-label period. After 52 weeks of treatment, ALI/AML sustained an acceptable safety profile for hypertensive patients, with a slight increase in AE incidence over time in the open-label period, consistent with other combination regimens with similar components [30, 31].

Hyperuricemia is commonly associated with hypertension and increases cardiovascular event risk [32]. Both ARBs and CCBs have been shown to effectively reduce uric acid levels [33]. In this study, the ALI/AML group had a greater reduction in uric acid levels (12.4 μmol/L) compared to the ALI group (8.6 μmol/L; conversion factor:1 mg/dL = 60 μmol/L). Although a trend toward reduced uric acid levels was evident, the impact was relatively small, likely attributable to the limited sample size. During the double-blind period, 1 patient (1.0%) in the AML/ALI group experienced hyperuricemia, which was not drug-related. In the open-label period, hyperuricemia occurred in 17 (9.4%), with only 4 (2.2%) considered study drug-related.

ARBs are recommended as first-line agents for the treatment of hypertension [14]. However, low-dose monotherapy may not suffice for adequate blood pressure control [34, 35]. Most hypertensive patients require at least two antihypertensive medications for optimal control [13–16]. SPCs enhance long-term treatment adherence, reduce pill burden, and improve blood pressure control, thereby lowering the risk of cardiovascular events [12]. The combination of ARB and CCB is a recommended optimized therapy in guidelines across China, America, and Europe [13–16]. with a synergistic antihypertensive effect.

This study confirmed the efficacy and safety of the ALI/AML combination but had several limitations. Firstly, the elderly population, who often face challenges in medication adherence and blood pressure control, was underrepresented. Future studies should focus on this demographic. Secondly, the patients with hypertension uncontrolled with ALI 240 mg were continued on the same dose of ALI 240 mg as comparator, without comparisons with higher doses of ALI or combination therapy(i.e., the addition of indapamide). However, the maximum dose of ALI approved was 240 mg; higher doses may be explored in future studies. Thirdly, The study population was more restricted than typical clinical practice, excluding those with poorly controlled diabetes or a BMI over 30 kg/m². Finally, the sample size for the exploratory ABPM analysis was small, potentially limiting the ability to detect significant differences between treatment groups. Future studies should include larger sample sizes to robustly evaluate the efficacy of ALI/AML in continuous blood pressure monitoring settings.

## Conclusion

ALI/AML demonstrated superior efficacy in reducing both office and ambulatory blood pressures in patients who showed an inadequate response to ALI monotherapy. The combination was well tolerated with once-daily dosing. The SPC of ALI/AML provides a convenient and effective therapeutic option for BP management in hypertensive patients.

## Key Learning Points

### What is known about the topic?

- Hypertension represents a crucial risk factor and exhibits a close association with the incidence and mortality of cardiovascular and cerebrovascular diseases.
- The combination of ARB + CCB is one of the recommended optimized combination therapy options in clinical practice in China, America and Europe.
- Single-pill combinations boost long-term treatment adherence and lessen pill burden. The single-pill combination of Allisartan and Amlodipine provides hypertensive patients with a convenient and effective method of reducing blood pressure in patients with essential hypertension uncontrolled by Allisartan Isoproxil.

### What this study adds

- For patients with essential hypertension uncontrolled with Allisartan Isoproxil, Allisartan Isoproxil/Amlodipine can further lower msSBP, being significantly superior to Allisartan Isoproxil monotherapy.
- Allisartan Isoproxil/Amlodipine has a good safety profile in patients with essential hypertension through once-daily administration.

## Supplementary information

Supplementary Methods and Supplementary Results

Supplementary Table

## Data Availability

There is no shared data for this study.
