# Supplementary material for: Efficacy and Safety of Allisartan Isoproxil/Amlodipine in Patients with Essential Hypertension: A Phase III, Multicenter, Double-Blind, Parallel-Group, Randomised study
Source: J Hum Hypertens. 2025 Jun 17;39(7):500–8. doi: 10.1038/s41371-025-01035-3 (PMC12343297; doi:10.1038/s41371-025-01035-3)
Supplement: Supplementary file 1 — Supplementary Methods and Supplementary Results [file 41371_2025_1035_MOESM1_ESM.docx]

**Supplementary Methods**

**Inclusion criteria**

1. Male or female patients aged 18 to 70 years (inclusive).
2. Body mass index (BMI) between 18.5 and 30 kg/m² (calculated as weight in kg/height in m²).
3. Patients diagnosed with primary hypertension according to the 2018 Revised Edition of the Chinese Guidelines for Hypertension Prevention and Treatment.
4. At the time of screening, patients must meet at least one of the following conditions: A. Untreated patients: Including those newly diagnosed or with a history of hypertension who have not taken antihypertensive medication for at least 2 weeks prior to screening, with mean sitting systolic blood pressure (msSBP) between 150 and < 180 mmHg and mean sitting diastolic blood pressure (msDBP) < 110 mmHg. B. Patients on irregular treatment: Patients not regularly taking Allisartan Isoproxil（240mg/day） for less than 4 weeks before screening, or with missed doses ≥ 5 days within the last 4 weeks, with msSBP between 140 and < 180 mmHg and msDBP < 110 mmHg. C.Patients on other antihypertensive treatments: Those taking non-study antihypertensive medications for at least 2 weeks before screening, with msSBP between 140 and < 180 mmHg and msDBP < 110 mmHg, deemed suitable by the clinician to switch to Allisartan Isoproxil（240mg/day）. D. Patients receiving stable treatment: Those who have been receiving stable treatment with Allisartan Isoproxil（240mg/day）for at least 4 weeks, with msSBP between 140 and < 180 mmHg and msDBP < 110 mmHg.
5. At randomization during the double-blind treatment phase, the msSBP must be between 140 and < 180 mmHg and msDBP < 110 mmHg.
6. Willingness of the participant (and their partner) to use effective contraception from screening through 6 months after the last dose of the study drug.
7. Medication compliance rate between 80% and 120% (inclusive) before randomization into the double-blind treatment phase.
8. Ability to understand and willingness to sign the informed consent form.
9. For participants undergoing ambulatory blood pressure monitoring (ABPM), the following additional criterion must be met: ABPM participants must have a 24-hour mean ambulatory BP ≥ 130/80 mmHg after 4 weeks of monotherapy. (Patients not meeting this criterion may still participate in the non-ABPM portion of the study.)

**Exclusion criteria**

1. Patients with secondary hypertension (e.g., renal hypertension, Cushing's syndrome, primary aldosteronism, pheochromocytoma, drug-induced hypertension).
2. Patients with msSBP of ≥ 180 mmHg and/or msDBP ≥ 110 mmHg or those experiencing acute or subacute hypertensive crises.
3. Known allergy or intolerance to calcium channel blockers (CCBs), angiotensin receptor blockers (ARBs), Amlodipine, or Allisartan (e.g., angioedema).
4. Concurrent use of more than two antihypertensive medications (including combination preparations) within 1 month prior to screening.
5. History of heart failure (New York Heart Association Class III or IV), acute coronary syndrome, percutaneous coronary intervention, or other severe cardiac conditions (e.g., cardiogenic shock, moderate to severe valvular heart disease, second- or third-degree atrioventricular block, bradycardia [heart rate < 50 bpm], severe arrhythmias) within the past 6 months.
6. History of severe cerebrovascular disease (e.g., hypertensive encephalopathy, cerebrovascular injury, stroke, transient ischemic attacks) within the past 6 months.
7. Presence of large aneurysms, aortic dissection, or dissecting aneurysms.
8. History of **severe** gastrointestinal disease or recent gastrointestinal surgery (within the past 3 months) that may interfere with drug absorption or metabolism (e.g., gastrointestinal resection, active ulcer, gastrointestinal bleeding).
9. History of malignancy within 5 years prior to screening.
10. Poorly controlled diabetes (fasting blood glucose ≥ 11 mmol/L).
11. Renal artery stenosis or severe renal insufficiency (serum creatinine > 1.5 times the upper limit of normal).
12. Serum potassium > 5.5 mmol/L.
13. Active viral hepatitis (including hepatitis B and C), other serious liver diseases, or hepatic dysfunction (alanine aminotransferase [ALT] or aspartate aminotransferase [AST] > 2.5 times the upper limit of normal, total bilirubin [TBIL] > 2 times the upper limit of normal).
14. Positive tests for HIV antibodies or Treponema pallidum-specific antibodies.
15. History of blood donation or significant blood loss (> 400 mL) within 3 months prior to screening or clinical diagnosis of hypovolemia.
16. Female participants who are breastfeeding or have a positive serum pregnancy test at screening.
17. History of substance abuse or alcoholism within 6 months prior to screening (defined as consumption of more than 14 units of alcohol per week; 1 unit = 285 mL beer, 25 mL spirits, or 100 mL wine).
18. Participation in another drug or device clinical trial within 3 months prior to screening.
19. Patients whom the investigators deem unsuitable for participation in the clinical trial.

**Supplementary Results**

**Supplementary Table 2**: Clinical centers and investigators participating in the study

| Center role | Center | Investigator |
| --- | --- | --- |
| Leading center | Beijing Chaoyang Hospital, Capital Medical University | Xinchun Yang (Principal Investigator)  Xiangmin Lin |
| Participating center | Beijing Tongren Hospital, Capital Medical University | Guohong Wang |
|  | Songgang People's Hospital, Bao'an District, Shenzhen City | Suolong Zhang |
|  | Shenzhen People's Hospital | Shaohong Dong |
|  | The University of Hong Kong-Shenzhen Hospital. | Mingya Liu |
|  | The Fifth Affiliated Hospital of Sun Yat-sen University | Xiufang Lin |
|  | The First Affiliated Hospital of Bengbu Medical University | Ningru Zhang |
|  | Chongqing General Hospital | Minfeng Li |
|  | Chongqing University Three Gorges Hospital | Huaming Mou |
|  | The First Affiliated Hospital of Fujian Medical University | Jinxiu Lin |
|  | Cangzhou Central Hospital | Jun Zhang |
|  | The Fourth Hospital of Hebei Medical University | Xingtao Li |
|  | The First Hospital of Hebei Medical University | Gang Liu |
|  | Xingtai People's Hospital | Qingmin Wei |
|  | The First Affiliated Hospital of Henan University of Science and Technology | Xuming Yang |
|  | Puyang Oilfield General Hospital | Hengliang Wang |
|  | Yueyang Central Hospital | Xiping Xu/Gang Pan |
|  | The Third Xiangya Hospital of Central South University | Hong Yuan |
|  | China-Japan Union Hospital of Jilin University(The Third Bethune Hospital of Jilin University ) | Ping Yang |
|  | Yanbian University Hospital (Yanbian Hospital) | Xiang Li |
|  | The Affiliated Hospital of Xuzhou Medical University | Defeng Pan |
|  | The People's Hospital Of Liaoning Province | Zhanquan Li |
|  | Panjin Liaoyou Baoshihua Hospital | Liqun Liu |
|  | Shengjing Hospital of China Medical University | Shumei Ma |
|  | The First Affiliated Hospital of Baotou Medical College | Xin Zhang |
|  | Inner Mongolia People's Hospital | Yuan Zhang |
|  | West China Hospital, Sichuan University | Xiaoyang Liao |
|  | The Affiliated Hospital of Southwest Medical University | Zhongcai Fan |
|  | Heze Municipal Hospital | Jihua Liang |
|  | Central Hospital Affiliated to Shandong First Medical University | Guohai Su |
|  | Tianjin Medical University | Liping Wei |
|  | Tianjin Medical University General Hospital | Yuemin Sun |
|  | Zhejiang Provincial People's Hospital | Jianhong Xie |
|  | China-Japan Friendship Hospital | Yihong Sun |
|  | Shanghai Fengxian District Central Hospital | Zengyong Qiao |
|  | The First Affiliated Hospital of Jinan University | Shaorong Wu |
|  | The People's Hospital of Guangxi Zhuang Autonomous Region | Shaoming Qin |
|  | The Second Xiangya Hospital of Central South University | Ling Liu |
|  | Zhongda Hospital Southeast University | Qiming Dai |
|  | Xuzhou Central Hospital | Hongyun Ruan |
|  | Zigong Third People's Hospital | Yufang Liu |
|  | The Second Affiliated Hospital of Guilin Medical University | Fusheng Xie |
