# Supplementary material for: Efficacy and Safety of Allisartan Isoproxil/Amlodipine in Patients with Essential Hypertension: A Phase III, Multicenter, Double-Blind, Parallel-Group, Randomised study
Source: J Hum Hypertens. 2025 Jun 17;39(7):500–8. doi: 10.1038/s41371-025-01035-3 (PMC12343297; doi:10.1038/s41371-025-01035-3)
Supplement: Supplementary file 2 — Supplementary Table [file 41371_2025_1035_MOESM2_ESM.docx]

**Supplementary Table 1.** Biochemical laboratory data collected during the study (SS)

| Variable | ALI/AML (n=98) | | | | | ALI (n= 100) | | | | |
| --- | --- | --- | --- | --- | --- | --- | --- | --- | --- | --- |
|  | **Baseline** | **Week 12** | | **Week 52** | | **Baseline** | **Week 12** | | **Week 52** | |
|  | **Mean (SD)** | **Mean (SD)** | **Change from baseline (SD)** | **Mean (SD)** | **Change from baseline (SD)** | **Mean (SD)** | **Mean (SD)** | **Change from baseline (SD)** | **Mean (SD)** | **Change from baseline (SD)** |
| Serum alanine aminotransferase (U/L) | 23.15  (13.29) | 22.79  (11.18) | -0.67  (10.76) | 25.66  (15.96) | 2.14  (12.02) | 26.18  (15.42) | 24.48  (13.98) | -1.30  (12.24) | 26.15  (23.67) | 0.25  (24.25) |
| Serum aspartate aminotransferase (U/L) | 22.90  (7.77) | 22.10  (6.22) | -1.36  (6.26) | 23.42  (11.33) | 0.16  (8.72) | 23.39  (8.13) | 22.27  (7.56) | -1.05  (8.38) | 22.72  (8.28) | -0.72  (9.81) |
| Serum glucose (mmol/L) | 5.77  (0.94) | 5.88  (1.53) | 0.19  (1.12) | 5.86  (0.92) | 0.10  (0.81) | 6.14  (1.24) | 6.21  (1.52) | 0.10  (0.96) | 6.30  (1.87) | 0.18  (1.20) |
| Serum creatinine (µmol/L) | 68.23  (15.66) | 69.12  (16.67) | 0.72  (7.19) | 67.74  (16.43) | -0.31  (8.02) | 68.73  (15.62) | 68.13  (15.73) | 0.82  (6.40) | 68.47  (13.62) | 1.03  (7.01) |
| Serum total cholesterol (mmol/L) | 5.04  (1.08) | 5.06  (1.01) | -0.03  (0.84) | 4.89  (1.01) | -0.09  (0.81) | 4.95  (1.03) | 5.04  (0.98) | 0.16  (0.75) | 4.91  (0.93) | -0.01  (0.84) |
| Serum triglyceride (mmol/L) | 1.88  (1.31) | 2.00  (1.66) | 0.11  (1.47) | 1.98  (1.73) | 0.12  (1.61) | 1.63  (1.09) | 1.69  (1.19) | 0.05  (0.98) | 2.04  (2.01) | 0.42  (1.57) |
| Serum sodium (mmol/L) | 140.60  (2.12) | 140.61  (2.07) | 0.07  (2.23) | 140.83  (2.24) | 0.33  (2.30) | 140.70  (1.98) | 140.78  (2.10) | 0.15  (2.26) | 140.73  (2.30) | 0.02  (2.49) |
| Serum potassium (mmol/L) | 4.19  (0.35) | 4.23  (0.36) | 0.01  (0.36) | 4.24  (0.40) | 0.04  (0.39) | 4.24  (0.26) | 4.28  (0.31) | 0.05  (0.38) | 4.27  (0.34) | 0.02  (0.33) |

SS: safety set; SD: standard deviation.
